# Expression of the Grape *VqSTS21* Gene in *Arabidopsis* Confers Resistance to Osmotic Stress and Biotrophic Pathogens but Not *Botrytis cinerea*

**DOI:** 10.3389/fpls.2016.01379

**Published:** 2016-09-15

**Authors:** Li Huang, Songlin Zhang, Stacy D. Singer, Xiangjing Yin, Jinhua Yang, Yuejin Wang, Xiping Wang

**Affiliations:** ^1^State Key Laboratory of Crop Stress Biology in Arid Areas, College of Horticulture, Northwest A&F UniversityYangling, China; ^2^Key Laboratory of Horticultural Plant Biology and Germplasm Innovation in Northwest China, Ministry of Agriculture, Northwest A&F UniversityYangling, China; ^3^Department of Agricultural, Food and Nutritional Science, University of Alberta, EdmontonAB, Canada

**Keywords:** stilbene synthase, piceid, grapevine, powdery mildew, *Botrytis cinerea*, salt stress, drought stress, *Arabidopsis*

## Abstract

Stilbene synthase (*STS*) is a key gene in the biosynthesis of various stilbenoids, including resveratrol and its derivative glucosides (such as piceid), that has been shown to contribute to disease resistance in plants. However, the mechanism behind such a role has yet to be elucidated. Furthermore, the function of *STS* genes in osmotic stress tolerance remains unclear. As such, we sought to elucidate the role of *STS* genes in the defense against biotic and abiotic stress in the model plant *Arabidopsis thaliana*. Expression profiling of 31 *VqSTS* genes from *Vitis quinquangularis* revealed that *VqSTS21* was up-regulated in response to powdery mildew (PM) infection. To provide a deeper understanding of the function of this gene, we cloned the full-length coding sequence of *VqSTS21* and overexpressed it in *Arabidopsis thaliana* via *Agrobacterium*-mediated transformation. The resulting *VqSTS21 Arabidopsis* lines produced *trans*-piceid rather than resveratrol as their main stilbenoid product and exhibited improved disease resistance to PM and *Pseudomonas syringae* pv. *tomato* DC3000, but displayed increased susceptibility to *Botrytis cinerea*. In addition, transgenic *Arabidopsis* lines were found to confer tolerance to salt and drought stress from seed germination through plant maturity. Intriguingly, qPCR assays of defense-related genes involved in salicylic acid, jasmonic acid, and abscisic acid-induced signaling pathways in these transgenic lines suggested that *VqSTS21* plays a role in various phytohormone-related pathways, providing insight into the mechanism behind *VqSTS21*-mediated resistance to biotic and abiotic stress.

## Introduction

Plants are exposed to an environment that is suffused with multiple challenges, including numerous types of biotic and abiotic stresses. Fortunately, they have evolved the capability to resist such environmental attacks through the development of a series of elaborate and sensitive defense response mechanisms. One such mechanism comprises the production of phytoalexins, which are low molecular weight secondary metabolites that are produced by plants and have been found to provide some level of resistance to multiple pathogens (Jeandet et al., 2002). Indeed, they are often used as markers of plant disease resistance (Sbaghi et al., 1995).

Resveratrol (3, 5, 4′-trihydroxy-stilbene) is a stilbenoid phytoalexin that is produced in a small number of plant species, including grapevine, peanut and pine, and functions in the defense against invasion and injury from various microorganisms and environmental stresses (Jeandet et al., 2002). Intriguingly, it has been linked not only to enhanced plant disease resistance, but also exhibits myriad medicinal benefits, such as anti-inflammatory, antioxidant and anticancer properties (Aggarwal et al., 2004; Shankar et al., 2007), which has made it the focus of much research. The production of this compound relies upon the polyphenol biosynthetic pathway, with the stilbene synthase (STS) enzyme catalyzing the final step in its biosynthesis. This protein shares the same substrates as chalcone synthase (CHS), which is a key enzyme in the biosynthesis of numerous flavonoids, including anthocyanins (Tropf et al., 1994).

In addition to its many direct biological roles, resveratrol can also be utilized as a backbone for the downstream production of additional stilbenoids, such as piceid (a 3-β-glucoside derivative of resveratrol) (Halls and Yu, 2008). Like resveratrol, piceid has been demonstrated to enhance disease resistance to fungal pathogens in grape leaves (Hanawa et al., 1992), which suggests that these two compounds may have similar biological activities. Both resveratrol and piceid occur in *cis-* and *trans-* configurations, with the *trans-* isomers being the biologically active form.

In recent years, *STS* genes have been transformed into various different plant species, such as tomato (Thomzik et al., 1997), papaya (Zhu et al., 2004), *Arabidopsis* (Yu et al., 2006; Liu et al., 2011), and hop (Schwekendiek et al., 2007), in an attempt to investigate their role in disease resistance. Interestingly, while the resistance of the resulting transgenic lines to pathogens was enhanced to some extent in every case, fungal infections were often not completely eradicated (Thomzik et al., 1997) and instead disease symptoms were merely partially mitigated or delayed (Zhu et al., 2004; Yu et al., 2006; Liu et al., 2011). In addition, there is some evidence that the heterologous expression of *STS* may yield distinct resistance responses to different pathogens, as exemplified by transgenic tomatoes expressing *STS*, which exhibited resistance to *Alternaria solani* but not *Botrytis cinerea* (Thomzik et al., 1997). Although these results are intriguing, further investigation will be required to decipher the precise relationship between pathogen resistance and heterologous *STS* expression in terms of both mechanism and possible differential responses.

While the majority of previous reports have focused mainly on the involvement of *STS* and stilbenoids in disease resistance against pathogens in plants (Jeandet et al., 2002; Yu et al., 2006; Liu et al., 2011), a small number of studies have demonstrated that osmotic stress can also activate the expression of *STS* (Deis et al., 2011; Hatmi et al., 2014) and that tolerance to osmotic stress was improved in plants in which stilbenoids had accumulated (Dubrovina et al., 2013). Indeed, it has been shown that grapevine exposed to drought stress or transgenic grapevine overexpressing calcium-dependent protein kinases, which play a central role in plant adaptation and resistance to biotic stress and abiotic stress (Aleynova-Shumakova et al., 2014), cause up-regulation of the expression of *STS* and contribute to stilbenoid biosynthesis (Dubrovina et al., 2013). Although the mechanism behind such a role for plant stilbenoids has yet to be elucidated, it was recently demonstrated that these molecules reduce the accumulation of reactive oxygen species (ROS) (Chang et al., 2011), which typically cause damage in plants in response to osmotic stresses such as high salinity, heat, cold or drought, and provides further evidence that *STS* may be involved in abiotic stress tolerance. However, these latter studies have focused mainly on the ability of stilbenoids to reduce ROS damage in plants in response to UV irradiation (Yin et al., 2016); therefore, further investigation will be required to determine if this also holds true for osmotic stress.

Grapevine is one of the most economically important fruit crops worldwide (Ji and Wang, 2013) and produces relatively high levels of stilbenoids. However, despite considerable progress in grape research, there remain substantial gaps in our understanding of the biological activity of molecules such as resveratrol and piceid. Therefore, in an attempt to further our knowledge regarding the role of stilbenoids and *STS* in biotic and abiotic stress responses, we generated transgenic *Arabidopsis* lines that heterologously and constitutively expressed a grape *STS* gene (*VqSTS21*) from *Vitis quinquangularis* cv. ‘Shang-24’. We then assessed the resistance of transgenic lines following infection with three types of pathogenic fungi, respectively, including biotrophic powdery mildew (PM; *Golovinomyces cichoracearum* UCSC1), semi-biotrophic *Pseudomonas syringae* pv. *tomato* (*Pst*) DC3000, and necrotrophic *B. cinerea*, to determine whether differential responses were evident. In addition, we evaluated the response of transgenic *Arabidopsis* lines compared to untransformed controls when subjected to osmotic stress. Finally, since phytohormone signal transduction pathways, such as those involving salicylic acid (SA), jasmonic acid (JA), and abscisic acid (ABA), play such an important role in plant stress response, we also explored the relationship between *STS* expression and the expression of genes required in these pathways in order to provide insight into the mechanism behind *STS*-induced stress resistance. Our findings not only further our understanding of biotic and abiotic stress-resistance pathways in plants, but also impart a framework for the future amelioration of disease and stress-tolerance in grapevine.

## Materials and Methods

### Inoculation of Grape with Powdery Mildew

Fully expanded leaves from 2-year old Chinese wild *V. quinquangularis* cv. “Shang-24” were infected with PM through gentle contact with leaves exhibiting disease symptoms. Infected samples were subsequently collected at 0, 6, 12, 24, 36, 48, 72, and 96 hours post-inoculation (hpi). Control leaves were sprayed with sterile distilled water and were harvested at the same time points. Samples were immediately frozen in liquid nitrogen, and then stored at -80°C until subsequent RNA extraction.

### RNA Extraction and Semi-Quantitative Real-Time PCR of Grape Leaves

Total RNA was extracted from *V. quinquangularis* cv. “Shang-24” leaf samples inoculated with PM 0, 6, 12, 24, 36, 48, 72, and 96 hpi using the E.Z.N.A. ^®^Plant RNA Kit (Omega Bio-tek, Norcross, GA, USA). Subsequent first-strand cDNA synthesis was carried out using PrimerScript^TM^RTase according to the manufacturer’s instructions (TaKaRa Bio Inc., Dalian, China). Amplification of cDNA was conducted using 2× Taq PCR MaterMix (BioSci Biotech, Hangzhou, China) and primers specific to each of the 31 *VqSTS* transcripts, respectively (primer sequences are provided in Supplementary Table S1). In each case, reactions generated amplicons ranging in size from 77 to 670 bp. The grape *Actin1* gene (GenBank Acc. No. AY680701) was utilized as an internal reference. Thermal parameters for PCR amplification were as follows: 94°C for 2 min, followed by 30–40 cycles of 92°C for 30 s, 60 ± 5°C for 30 s, 72°C for 30 s, and final extension at 72°C for 2 min. PCR products were separated on a 1.5% (w/v) agarose gel and imaged under UV light. GeneSnap and HemI 1.0 (Deng et al., 2014) programs were utilized for assessing relative semi-quantitative expression levels. All reactions were carried out in triplicate.

### Generation of Transgenic Plants and Growth Conditions

The full-length 1170 bp *VqSTS21* coding sequence was amplified using gene-specific primers and 2× Taq PCR MasterMix (BioSci Biotech, Hangzhou, China). The resulting PCR product was then cloned into the pGEM^®^-T Easy vector (Promega, Madion, WI, USA). Restriction sites were added to the ends of the *VqSTS21* coding sequence in order to facilitate downstream cloning using gene-specific primers with *BamH* I and *Sma* I sites added to their 5′ termini, respectively. Following cloning, the *VqSTS21* coding sequence was inserted downstream of the CaMV 35S promoter in the plant expression vector pCambia 2300 (Cambia, Brisbane, QLD, Australia).

The resulting plant transformation vector was introduced into *A. thaliana* (Col-0) using the floral dip method (Clough and Bent, 1998). To identify transgenic lines, T_1_ seeds were harvested and sown on MS medium (PhytoTechnology Laboratories, Overland Park, KS, USA) containing 10 g/L sucrose, 8 g/L agar at pH 5.8 and supplemented with 75 mg/L kanamycin. Fifteen independent lines were found by segregation analysis of T_2_ seeds to bear a single copy of the transgene and were confirmed as transgenic using PCR (data not shown). Subsequently, these 15 lines were inoculated with PM and the three lines (L1, L2, and L3) exhibiting the highest level of disease resistance were selected for all further experiments. T_3_ homozygous L1, L2, and L3 lines were utilized for the remainder of this study and wild-type (Col-0) plants were used as the untransformed control. All *Arabidopsis* plants were grown at 21∼23°C with a 16 h/8 h photoperiod (100 μmol m^-2^ s^-1^ photon flux density) at ∼60% relative humidity (RH) on soil.

### *Arabidopsis* Pathogen Inoculation Assays

Powdery mildew (10–14 days post-inoculation, dpi) cultivated on 4-week old *pad4* (*phytoalexin deficient 4*) mutant *Arabidopsis* plants, which are very susceptible to this pathogen (Reuber et al., 1998), was utilized as inoculum to inoculate 24 four-week old Col-0 and transgenic plants, respectively. Treated plants were incubated at 22°C with 16 h light and ∼80% RH for 3 days following inoculation and were then transferred to an ambient environment at 22°C and 30∼40% RH. The response of *VqSTS21* transgenic plants and untransformed controls was monitored between 0 and 7 dpi.

*Botrytis cinerea* was obtained from tomato and cultured on potato dextrose agar medium at 25°C in the dark. Conidial spore suspensions (2 × 10^7^ conidia/ml) were prepared with 14-day old cultures using sterile, distilled water as described previously (Guo et al., 2016). Fifty leaves from 24 four-week old plants, including the three transgenic lines and untransformed controls, respectively, were rinsed with distilled water and 10 μl conidial suspension was applied, after which time the leaves were placed on 1% agarose overlaid with wet filter paper and the glass Petri dishes were then sealed. Inoculated leaves were incubated at 22°C with a 16/8 h light cycle and ∼95% RH. Disease incidence and lesion diameter were recorded daily until 4 dpi.

*Pst* DC3000 was grown at 28°C in King’s B medium (Tornero and Dangl, 2001) supplemented with 50 μg/ml rifampicin in a shaker at 280 rpm until it reached an OD_600_ of 0.8∼1.0. The resulting cell suspension was centrifuged at 5000 *g* for 10 min, and was then diluted with 10 mM MgCl_2_ to an OD_600_ of 0.02. Four-week old transgenic and Col-0 plants were dipped into the cell suspension containing 0.02% Silwet L-77 for 10 min as described previously (Tornero and Dangl, 2001), and were then covered with a plastic lid to maintain a high level of humidity for 24 h. Disease symptoms were assessed 3 dpi. For bacterial population assays, leaf disks (0.5 cm × 0.5 cm) were collected from 8 independent L1, L2, L3 lines and untransformed control plants, respectively, at 3 dpi. Disks were rinsed with sterile water three times, homogenized in 100 μl 10 mM MgCl_2_, and the solution was gradually diluted. One hundred microliters of the resulting diluted solution were then plated onto King’s B agar plates supplemented with 50 μg/ml rifampicin and 50 μg/ml kanamycin for 48 h at 28°C (Guo et al., 2016).

### HPLC Analysis of *VqSTS21* Transgenic Lines

The accumulation of resveratrol and its stilbenoid derivates were analyzed in triplicate in PM-inoculated and mock-inoculated leaf samples (0.5 g each, 7 dpi) harvested from 4-week old *VqSTS21* transgenic lines and untransformed controls. The resulting samples were ground to a fine powder in liquid nitrogen using a mortar and pestle. Ground leaves were extracted with 5 ml 80% methanol, and supernatant fractions were collected following centrifugation at 4500 *g* for 5 min. The resulting extracts were evaporated using a vacuum rotary evaporator (CS110-4, LaboGene, Denmark), and were immediately re-dissolved in 0.2 ml of pure methanol. These extracts (30 μl) were then filtered through a 0.45 μm sterile Durapore^®^ PVDF filter (Millipore, USA). Samples were run on an Agilent 1200 HPLC system (Agilent, Waldbronn, Germany) with an Agilent ZORBAX SB-C18 column (5 μm, 4.6 × 250 mm), H_2_O-methanol as eluent (H_2_O:methanol [60:40], flow rate 0.8 ml/min), and a wavelength of 306 nm for detection. The column temperature was maintained at room temperature. Stilbenoids in transgenic lines were identified by comparing the retention time with those of standards.

### Histochemical Detection of Cell Death and Superoxide Accumulation

Superoxide anions ($O_{2}^{–}$) accumulation and cell death were monitored in 30 detached leaves from 12 transgenic and untransformed control plants, respectively, following pathogen inoculation using nitro blue tetrazolium (NBT) and trypan blue staining. Experiments involving PM were conducted 5 dpi while those involving *B. cinerea* and *Pst* DC3000 were carried out 3 dpi. In the case of NBT staining, inoculated leaves were incubated in HEPES buffer (pH 7.5) containing 6 mM NBT for 2∼3 h (Tu et al., 2016). Trypan blue staining was carried out as described previously (Guo et al., 2016). Briefly, infected leaves were soaked in boiled trypan blue solution (a 1:1:1:1:1 ratio of trypan blue, phenol, glycerol, lactic acid, and water) for 2–3 min, and were subsequently depigmented in 2.5 g/ml chloral hydrate for 1–2 days.

### Determination of Cotyledon Greening Rates Following Salt and Drought Stress

T_3_ seeds from transgenic lines and Col-0 plants were vernalized at 4°C for 3 days, disinfected in 75% ethanol for 30 s, washed with sterilized distilled water three times, incubated in 10% NaClO for 5 min, and finally washed with sterilized distilled water five times. To determine the inhibitory concentrations of NaCl and mannitol for cotyledon greening rates, both transgenic and Col-0 seeds were sown on MS medium containing different concentrations of NaCl and mannitol, respectively. Subsequently, 120 sterilized seeds from each line were sown on MS medium supplemented with 130 mM NaCl or 250 mM mannitol to stimulate salt and drought stress, respectively (Guo et al., 2016; Tu et al., 2016), and were grown at 21∼23°C with a 16 h/8 h photoperiod (100 μmol m^-2^ s^-1^ photon flux density). Cotyledon greening rates were recorded daily. Each experiment was conducted in triplicate.

### Effect of Osmotic Stress on Transgenic *Arabidopsis*

Fifteen 4-day old transgenic and Col-0 seedlings, respectively, that had been cultivated on MS medium were transferred to either fresh MS medium or MS medium supplemented with 130 mM NaCl, 250 mM mannitol or 0.75 μM ABA. Root lengths were measured 6 days following osmotic stress treatment.

To assess the response of mature plants to osmotic stress, 4-week old transgenic lines and Col-0 plants were treated with salt or drought. In the case of salt treatment, soil was allowed to dry somewhat prior to watering with 130 mM NaCl to prevent dilution, and watering with salt solution was carried out once every 3 or 4 days for 7 days. For drought treatment, watering was halted for 7 days. Performance of the plants was assessed and survival rates recorded 7 days following both types of osmotic treatment. In each instance, experiments were carried out in triplicate.

### Determination of Chlorophyll and MDA Contents, As Well As Relative Electrolyte Leakage, in Transgenic Seedlings

*VqSTS21* transgenic lines and Col-0 untransformed controls were sown on MS medium plates, and 1-week old seedlings were subsequently transferred to flasks containing MS liquid medium supplemented with 130 mM NaCl or 250 mM mannitol. Seven days following commencement of osmotic stress treatment, seedlings were collected for physiological assessments. To measure chlorophyll content, 0.1 g seedlings with their roots removed were submerged in 5 ml 96% ethanol and incubated at 4°C until the seedlings turned white (Guo et al., 2015).

For assessment of relative electrolyte leakage, 0.1 g seedlings were incubated in ultrapure water under vacuum for 20 min and were then left at room temperature for 2 h. Conductivity (C1) of the incubation solution was subsequently measured using a conductivity detector (FE30, METTLER-TOLEDO, China). Seedlings were then submerged in boiling ultrapure water for 20 min, cooled to room temperature, and the conductivities of the resulting solutions (C2) were once again determined. The values of relative electrolyte leakage were calculated using the ratio of C1–C2 (Bajji et al., 2002).

To determine malondialdehyde (MDA) content, 0.5 g seedlings were ground with 5 ml trichloroacetic acid in a mortar and then transferred to 10 ml centrifuge tubes for centrifugation at 4,000 rpm for 10 min. Subsequently, 2 ml of the supernatant was combined with 2 ml 0.67% (w/v) thilbarbituric acid, heated to 100°C for 15 min, immediately cooled on ice, and centrifuged at 4,000 rpm for 5 min. Finally, the absorbance of the supernatant was measured at 532 nm (A532), 600 nm (A600), and 450 nm (A450) using a spectrophotometer (UV-1700, Shimadzu, Japan). MDA content was calculated as follows: MDA content = 6.45 (A532- A600)-0.56 × A450 (Tu et al., 2016).

### Measurement of Water Loss Rates in Transgenic Plants

Five 4-week old *VqSTS21* transgenic L1, L2, and L3 lines, as well as Col-0 untransformed controls, respectively, were removed from soil and weighed immediately to determine the initial fresh weight of the plants. The plants were subsequently weighed every 15 min at room temperature in ambient conditions of approximately 40% RH. The experiment was carried out in triplicate.

### qPCR Analysis of Gene Expression

Leaves were harvested from L1, L2, L3 and Col-0 plants at 0, 24, 72, 120, and 168 hpi (PM infection), 0, 24, 48, 72, and 96 hpi (*B. cinerea* infection), 0, 24, and 48 hpi (*Pst* DC3000 infection), or at 0 and 7 dpi (osmotic stress treatment). Three biological replicates were collected from each line at each time point. Total RNA was extracted using the Ultrapure RNA kit (ComWin Biotech, Beijing, China), and first-strand cDNA synthesis was carried out using TransScript^®^ (Transgene Biotech, Beijing, China) in a reaction volume of 20 μl, which included 200 ng total RNA, 1 μl gDNA Remover and 1 μl Anchored Oligo (dT)_18_ Primer (0.5 μg/μl).

Subsequent quantitative real-time PCR assays were performed in triplicate in a final reaction volume of 20 μl, which included 1 μl of a sixfold cDNA dilution as template and 2× TransStart Tip Green qPCR Supermix (Transgene Biotech, Beijing, China). Gene-specific primers used in the reactions are listed in Supplementary Table S2. Assays were carried out using a CFX96 real-time PCR detection system (Bio-Rad, CFX96, USA) with thermal parameters of 94°C for 30 s, followed by 40 cycles of 94°C for 5 s and 60°C for 30 s. The *A. thaliana Actin2* (TAIR: AT3G18780) gene was used as an internal reference gene (Tu et al., 2016).

## Results

### Expression Profiles of Grape *VqSTS* Genes in Response to Infection by Powdery Mildew

**FIGURE 1:**
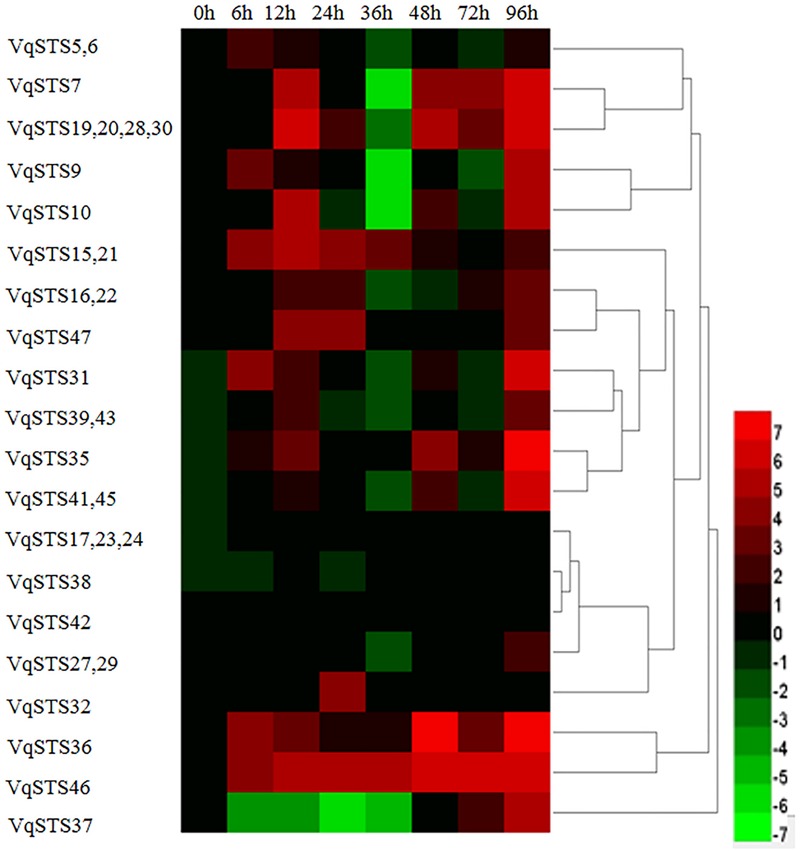
**Expression profiles of 31 grape *VqSTS* genes in response to powdery mildew infection.** Two-year old *V. quinqangularis* cv. “Shang-24” was inoculated with powdery mildew (PM), and leaves were collected 0, 6, 12, 24, 36, 48, 72, and 96 hpi for the analysis of gene expression via semi-quantitative real-time PCR. Leaves sprayed with water were used as untreated controls. The color scale represents the relative expression levels of each gene in response to PM infection, with red indicating increased transcript abundance and green depicting decreased transcript abundance compared to untreated controls. The experiment was carried out three times, with consistent results obtained in every case.

To obtain insight into the potential roles of all 31 *VqSTS* genes in the grape genome in terms of disease resistance, we inoculated 2-year old seedlings of *V. quinquangularis* cv. “Shang-24” with PM and assessed *STS* expression via semi-quantitative real-time PCR. Ten of the *VqSTS* genes analyzed demonstrated significant alterations in their expression levels following challenge with this pathogen (**Figure 1**), including *VqSTS19, VqSTS20, VqSTS28, VqSTS30, VqSTS15, VqSTS21, VqSTS36, VqSTS46*, and *VqSTS47*. Among these genes, the expression levels of *VqSTS15* and *VqSTS21* increased until reaching a peak at 12 hpi, which was earlier than other genes, and then declined at subsequent time points (**Figure 1**). As such, we selected the *VqSTS21* gene for all further functional analyses in this study.

### Heterologous Expression of *VqSTS21* in *Arabidopsis* Enhances Powdery Mildew Disease Resistance by Inducing the Salicylic Acid-Dependent Signaling Pathway

**FIGURE 2:**
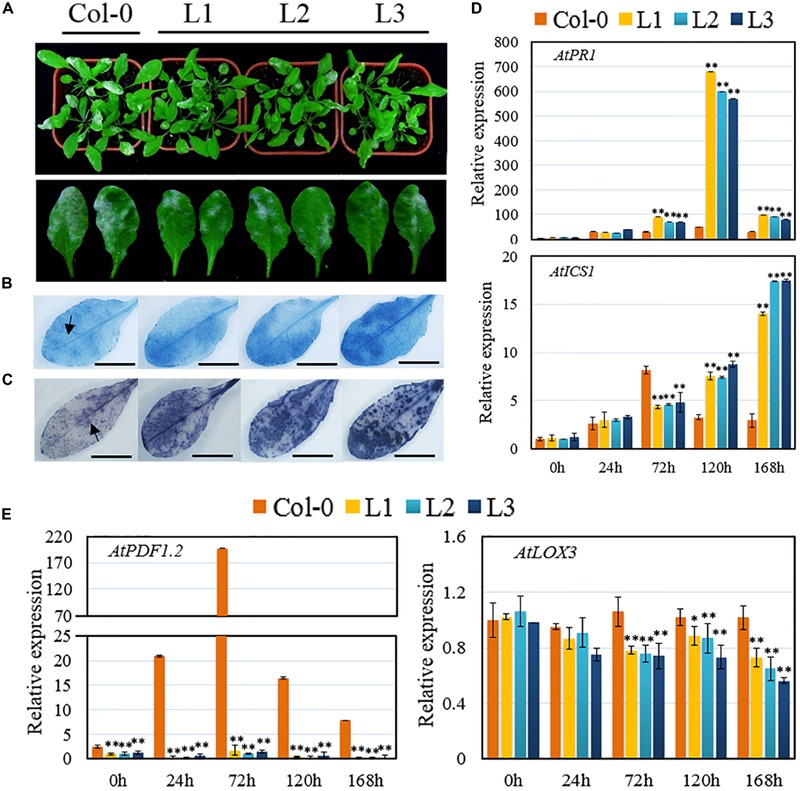
**Performance of *VqSTS21* transgenic *Arabidopsis* lines and untransformed controls following inoculation with powdery mildew and expression of defense-related genes.** *VqSTS21* transgenic lines (L1, L2, and L3) and untransformed controls (Col-0) were infected with powdery mildew (PM). **(A)** Representative images of plants were taken 7 days post-inoculation (dpi). Scale bar = 50 mm. **(B,C)** Trypan blue and nitro blue tetrazolium (NBT) staining were carried out on leaves 5 dpi in order to detect cell death **(B)** and superoxide anion ($O_{2}^{–}$) accumulation **(C)**, respectively, as noted by arrows. Scale bars = 10 mm. **(D,E)** Relative expression levels of defense-related genes in leaves collected 0, 24, 72, 120, and 168 h post-inoculation (hpi) as established via qPCR. Data represent mean values ± SD (with values from time point 0 hpi set to 1) from three independent experiments. Asterisks indicate significant differences between Col-0 and transgenic lines as determined by Student’s *t*-test. (^∗^0.01 < *P* < 0.05; ^∗∗^*P* < 0.01).

Transgenic *Arabidopsis* lines constitutively expressing the *VqSTS21* gene, along with untransformed plants, were inoculated with PM and assessed 7 dpi. Leaf surfaces of transgenic lines showed fewer disease symptoms than untransformed controls at 7 dpi (**Figure 2A**). Staining with trypan blue (**Figure 2B**) and NBT (**Figure 2C**) revealed that transgenic lines exhibited higher levels of cell death and accumulated more superoxide anions ($O_{2}^{–}$), respectively, than untransformed plants as evidenced by increased staining.

To obtain deeper insight into the pathway(s) driving these alterations in *VqSTS21* transgenic plants in response to PM, we assessed the relative expression levels of several SA- (**Figure 2D**) and JA-responsive genes (**Figure 2E**) in 4-week old plants at 0, 24, 72, 120, 168 hpi, respectively. qPCR assays indicated that pathogenesis-related gene 1 (*AtPR1*) and isochorismate synthase 1 (*AtICS1*), which play major roles in the SA-dependent disease resistance response, were significantly up-regulated following inoculation with PM in untransformed control plants (**Figure 2D**). In transgenic plants subjected to PM inoculation, expression of *AtPR1* was significantly up-regulated 12-fold compared to untransformed controls from 72 hpi onward. Similarly, while *AtICS1* expression was initially down-regulated at 72 hpi compared to untransformed controls, 2.4- and 5.5-fold increases in expression compared to untransformed plants were observed at 120 and 168 hpi, respectively (**Figure 2D**).

Conversely, although expression of plant defensin1.2 (*AtPDF1.2*) and lipoxygenase-3 (*AtLOX3*), which is key components of the JA-mediated signaling pathway, were also enhanced following PM inoculation in both untransformed and transgenic plants, their expression levels in transgenic plants were significantly reduced compared to untransformed controls. In particular, the expression of *AtPDF1.2* was decreased by 45.8-fold in transgenic lines compared to untransformed plants (**Figure 2E**).

### *Trans*-Piceid Is the Main Stilbenoid Produced in Transgenic Lines and Its Content Is Affected by Powdery Mildew Infection

Four-week old transgenic *STS* and untransformed plants were inoculated with PM, leaves were collected 7 dpi, and stilbenoid content was measured using HPLC. Untransformed control plants were not found to produce stilbenoids following infection, which agrees with previous studies (Yu et al., 2006; Liu et al., 2011). However, since *Arabidopsis* contains all of the substrates and enzymes required for stilbenoid production, with the exception of *STS*, transgenic lines expressing *VqSTS21* were found to produce an abundance of stilbenoids. Interestingly, the vast majority of the stilbenoids produced in these plants was in the form of *trans*-piceid rather than resveratrol (Supplementary Figure S1). In addition, *trans*-piceid content in transgenic lines infected with PM was found to be 1.3-fold higher than un-inoculated transgenic lines (**Table 1**).

**Table 1:** *Trans*-piceid content in transgenic lines inoculated with powdery mildew (PM) and untreated controls.

Treatment	Content of *trans*-piceid (μg g^-1^ of fresh weight)			
	Col-0	L1	L2	L3
CK	0	216.30 ± 16.18	414.60 ± 6.66	531.15 ± 13.23
Infected with PM	0	328.75 ± 8.76	541.10 ± 12.81	661.27 ± 10.59

*CK indicates un-inoculated plants; data represent mean values ± SD from three independent experiments.*

### *VqSTS21* Transgenic Lines Exhibit Increased Susceptibility to *B. cinerea* and Induce the Salicylic Acid-Dependent Signaling Pathway

**FIGURE 3:**
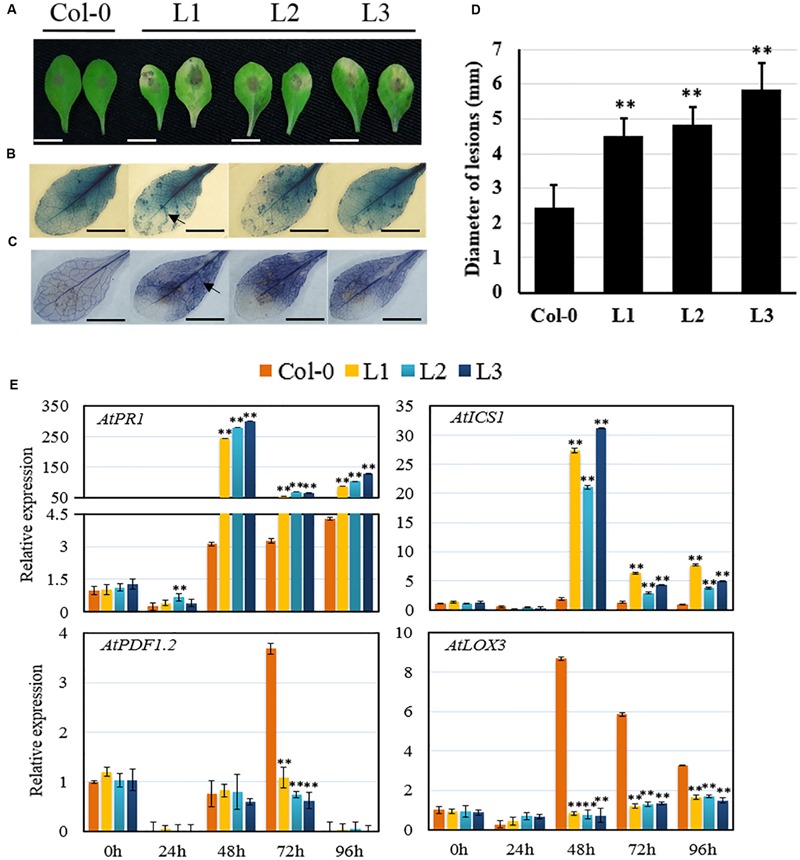
**Performance of *VqSTS21* transgenic *Arabidopsis* lines and untransformed controls following inoculation with *Botrytis cinerea* and expression of defense-related genes.** Leaves from transgenic lines (L1, L2, and L3) and untransformed controls (Col-0) were infected with *B. cinerea* spores. **(A)** Representative images of disease symptoms on leaves 4 days post-inoculation (dpi). **(B,C)** Trypan blue and nitro blue tetrazolium (NBT) staining were carried out 3 dpi in order to detect cell death **(B)** and superoxide anion ($O_{2}^{–}$) accumulation **(C)**, respectively, as noted by arrows. **(D)** *B. cinerea* lesion diameter at 4 dpi. **(E)** Relative expression levels of defense-related genes in leaves collected 0, 24, 48, 72, and 96 h post-inoculation (hpi) as established via qPCR. Data represent mean values ± SD (with values from time point 0 hpi set to 1) from three independent experiments. Asterisks indicate significant differences between Col-0 and transgenic lines as determined by Student’s *t*-test (^∗∗^*P* < 0.01). Scale bars = 10 mm.

To determine whether *VqSTS21* induces differential responses to particular pathogens, we assessed transgenic lines for their susceptibility to the necrotrophic fungal pathogen, *B. cinerea*. Three days following inoculation with the pathogen, transgenic plants exhibited more severe disease lesions than untransformed controls (**Figure 3A**), with a significant increase in lesion diameter (**Figure 3D**). Furthermore, histochemical assays indicated that transgenic lines displayed enhanced cell death (**Figure 3B**) and superoxide anion ($O_{2}^{–}$) production (**Figure 3C**) compared to untransformed plants.

Expression levels of defense-related genes were also assessed in response to *B. cinerea* inoculation using qPCR 0, 24, 48, 72, and 96 hpi, respectively (**Figure 3E**). Transcript levels of all four genes tested, including *AtPR1* and *AtICS1*, which are key components of SA-mediated defense signaling, as well as *AtPDF1.2* and *AtLOX3*, which are components of JA-mediated defense signaling, increased by 48 hpi in untransformed controls inoculated with *B. cinerea* (**Figure 3E**). From 48 hpi onward, transgenic lines expressing *VqSTS21* displayed significant up-regulation of *AtPR1* and *AtICS1*, with up to 87.9-fold and 14.4-fold increases in transcript levels, respectively, compared to untransformed controls. Conversely, *AtPDF1.2* and *AtLOX3* were significantly down-regulated from 72 and 24 hpi onward, respectively, in transgenic plants compared to untransformed controls (**Figure 3E**).

### *VqSTS21* Enhances Resistance to *Pst* DC3000 and Involves Both Salicylic Acid and Jasmonic Acid Signaling Pathways

**FIGURE 4:**
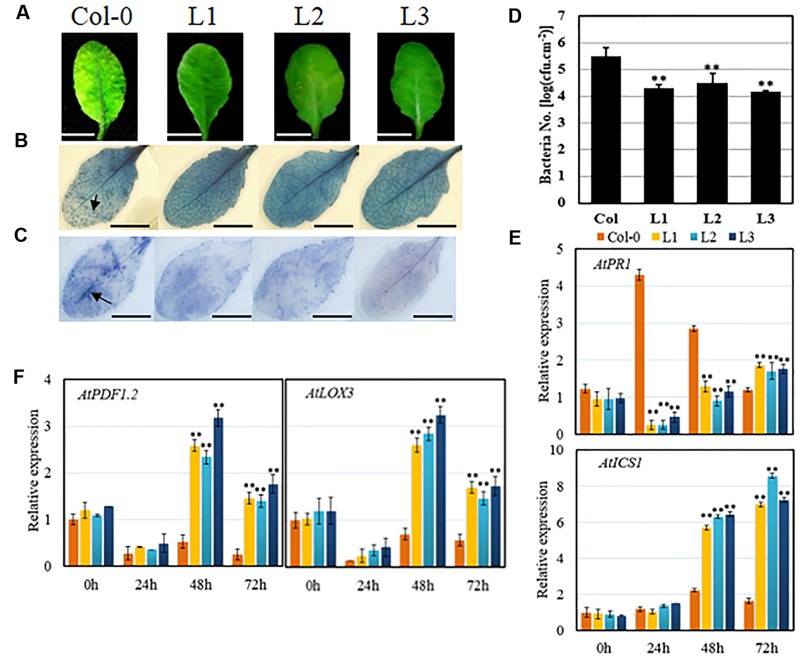
**Performance of *VqSTS21* transgenic *Arabidopsis* lines and untransformed controls following inoculation with *Pst* DC3000 and expression of defense-related genes.** Transgenic lines (L1, L2, and L3) and untransformed controls (Col-0) were infected with *Pst* DC3000. **(A)** Representative images of disease symptoms at 3 days post-inoculation (dpi). **(B)** Cell death and superoxide anion **(C)** were detected by trypan blue and nitro blue tetrazolium (NBT) staining, respectively, at 3 dpi, as noted by arrows. **(D)** Bacterial population assays were performed at 3 dpi. **(E,F)** Relative expression levels of defense-related genes in leaves collected 0, 24, 48, and 72 h post-inoculation (hpi) as established via qPCR. Data represent mean values ± SD (with values from time point 0 hpi set to 1) from three independent experiments. Asterisks indicate significant differences between Col-0 and transgenic lines as determined by Student’s *t*-test (^∗∗^*P* < 0.01). Scale bars = 10 mm.

Transgenic lines and untransformed controls were inoculated with *Pst* DC3000 to investigate whether *VqSTS21* was able to enhance resistance to the pathogen. Three days following inoculation, disease symptoms, as evidenced by chlorosis, were found to be more severe in untransformed control plants than transgenic plants (**Figure 4A**). Indeed, bacterial population assays (**Figure 4D**) suggested that *VqSTS21* inhibited the development of *Pst* DC3000 in transgenic lines, resulting in significant reductions in bacterial numbers in transgenic lines compared to untransformed controls. In addition, as was the case with PM and *B. cinerea* infection, trypan blue (**Figure 4B**) and NBT staining ($O_{2}^{–}$) (**Figure 4C**) indicated reduced levels of cell death and accumulation of superoxide anions in response to *Pst* DC3000 infection in transgenic lines compared to untransformed controls.

We have previously ascertained that a distinction can be made in gene expression levels within 72 h of inoculation with this pathogen (Guo et al., 2016). Therefore, expression levels of defense-related genes were assessed in response to *Pst* DC3000 inoculation using qPCR 0, 24, 48, and 72 hpi, respectively (**Figure 3E**). In a similar fashion to PM and *B. cinerea* infection, expression levels of *AtPR1, AtICS1, AtPDF1.2* and *AtLOX3* (**Figures 4E,F**) were all found to be up-regulated following inoculation with *Pst* DC3000 by 48 hpi in untransformed controls. In transgenic lines, the JA-mediated signaling-related genes, *AtPDF1.2* and *AtLOX3*, exhibited significant up-regulation compared to untransformed controls following inoculation from 48 hpi onward. Similarly, the SA-mediated signaling gene *AtICS1* demonstrated a significant increase in transcript levels compared to untransformed controls by 48 hpi. Conversely, the remaining SA-mediated signaling gene tested, *AtPR1*, exhibited a significant reduction in transcript levels compared to untransformed controls at both 24 and 48 hpi, but not 72 hpi.

### The Ability of Transgenic *Arabidopsis* Lines to Withstand Osmotic Stress Is Enhanced Compared to Untransformed Controls

**FIGURE 5:**
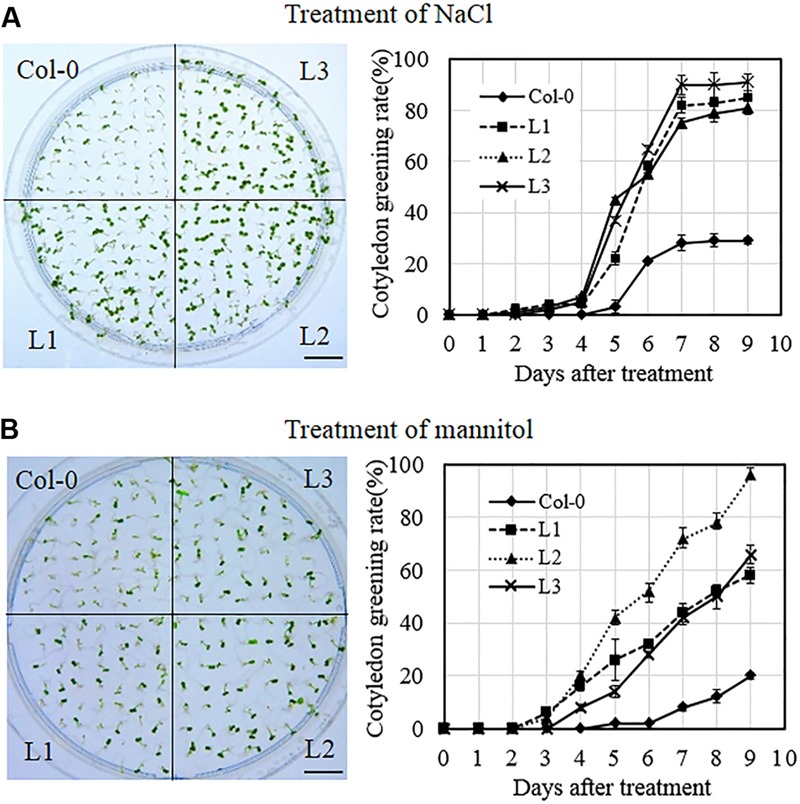
**Cotyledon greening rates of *VqSTS21* transgenic *Arabidopsis* lines following the induction of osmotic stress.** Homozygous seeds from *VqSTS21* transgenic lines (L1, L2, and L3) and untransformed controls (Col-0) were plated on MS media supplemented with 130 mM NaCl **(A)** and 250 mM mannitol **(B)**, respectively, and assessed for cotyledon greening rates. Representative images of plants were taken 12 days after seeds were plated on osmotic stress-inducing medium. Cotyledon greening rates were assessed daily following initiation of each treatment. Data represent mean values from three independent experiments. Scale bars = 10 mm.

To investigate the response of *VqSTS21* transgenic lines to osmotic stress, transgenic and Col-0 plants were exposed to salt and drought stress at three stages of development, including seeds, seedlings and mature plants. In the case of seeds, transgenic lines and Col-0 plants were grown on MS medium supplemented with 130 mM NaCl and 250 mM mannitol to simulate salt and drought stress, respectively, and rates of cotyledon greening in transgenic lines were found to be significantly higher than those of Col-0 seeds following both treatments (**Figures 5A,B**). This suggests that transgenic seeds acquired less damage from salt and drought stress than untransformed controls, and that *VqSTS21* expression enhanced the tolerance of *Arabidopsis* seeds to osmotic stress.

**FIGURE 6:**
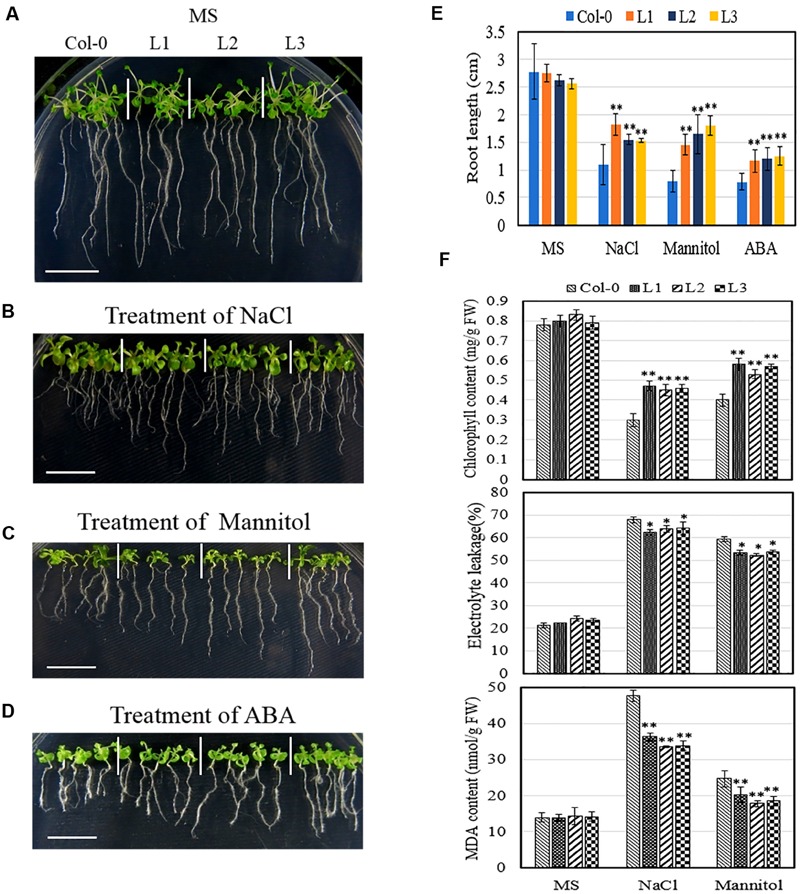
**Effect of osmotic stress on seedlings of *VqSTS21* transgenic *Arabidopsis* seedlings and untransformed controls.** Five-day old transgenic (L1, L2, and L3) and untransformed seedlings were transferred from MS plates to new MS plates **(A)**, MS supplemented with 130 mM NaCl **(B)**, 250 mM mannitol **(C)**, or 0.75 μM ABA **(D)**. Representative images were taken and root lengths were measured **(E)** 6 days after the initiation of osmotic stress treatment. Seven-day old *VqSTS21* transgenic (L1, L2, and L3) and untransformed (Col-0) seedlings were transferred from unamended MS medium to MS medium supplemented with 130 mM NaCl, 250 mM mannitol or 0.75 μM ABA, and physiological parameters were assessed 7 days following the initiation of osmotic stress treatments. Chlorophyll content, relative electrolyte leakage and malondialdehyde (MDA) content were measured in plants subjected to stress and untreated controls **(F)**. Data represent mean values ± SD from three independent experiments. Asterisks indicate significant differences between Col-0 and transgenic lines as determined by Student’s *t*-test (^∗^0.01 < *P* < 0.05; ^∗∗^*P* < 0.01). Scale bars = 10 mm.

In the case of seedlings, we assessed various physiological parameters to determine whether transgenic plants were better able to tolerate osmotic stress than untransformed controls. Since both root development and nutrient uptake are known to be negatively impacted by osmotic stress and high concentrations of ABA (de Dorlodot et al., 2007; Rowe et al., 2016), we first sought to evaluate the effect of *VqSTS21* overexpression in *Arabidopsis* on root development. Obvious differences were noted between *VqSTS21* transgenic seedlings and Col-0 seedlings following osmotic stress treatment (**Figures 6A–D**). Indeed, root lengths of transgenic seedlings subjected to 130 mM NaCl, 250 mM mannitol or 0.75 μM ABA were found to be significantly longer than those of Col-0 (**Figure 6E**), which indicates that they are capable of better tolerating osmotic stress than wild-type plants (Tardieu, 2012). To investigate the degree to which leaves were damaged by osmotic stress and whether the enhanced osmotic stress tolerance seen in transgenic lines is related to membrane permeability, chlorophyll content and relative electrolyte leakage assays were conducted, respectively (Bajji et al., 2002; Zhang et al., 2012; Tu et al., 2016). The chlorophyll content of transgenic seedlings was found to be significantly higher following salt (0.46 mg/g FW) and drought (0.56 mg/g FW) treatment than Col-0 (0.3 and 0.4 mg/g FW, respectively) plants. Electrolyte leakage was also significantly reduced in transgenic lines compared to untransformed controls following both types of osmotic stress (**Figure 6F**), which suggests that transgenic lines possess enhanced cell membrane integrity that could contribute to their ability to better withstand osmotic stress. Water deficit and high salinity are often associated with the production of MDA in plants, which causes membrane damage and cell death (Levine et al., 1994). In this study, we found MDA content to be significantly reduced in transgenic lines compared to untransformed controls following osmotic stress treatment (**Figure 6F**); a factor that could also play a role in osmotic stress resistance in these lines.

**FIGURE 7:**
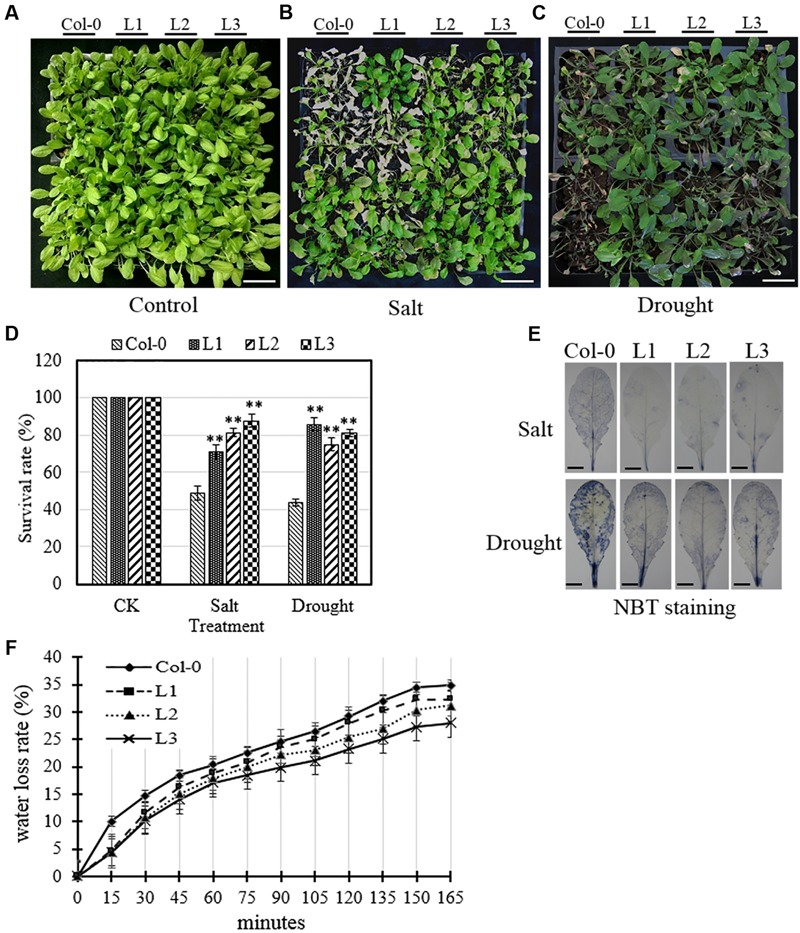
**Analysis of osmotic stress tolerance in *VqSTS21* transgenic *Arabidopsis* lines and untransformed controls.** Mature *VqSTS21* transgenic lines (L1, L2, and L3) and untransformed controls (Col-0) were subjected to salt stress (watering with 130 mM NaCl) or drought. **(A–C)** Representative images of 5-week old plants 7 days following no treatment **(A)**, or treatment with salt **(B)** and drought **(C)**, respectively. Scale bars = 40 mm. **(D)** Survival rates of plants were assessed 7 days following the initiation of salt and drought treatments. Asterisks indicate significant differences between Col-0 and transgenic lines as determined by Student’s *t*-test (^∗∗^*P* < 0.01). **(E)** Nitro blue tetrazolium (NBT) staining for detecting the accumulation of superoxide ($O_{2}^{–}$) was carried out using detached leaves from untreated and osmotic stress-treated plants. Scale bars = 3.5 mm. **(F)** The water loss rate of detached leaves from 5-week-old plants was determined in untreated plants.

In the case of mature plants, we found that the leaves of untransformed control plants displayed substantial amounts of chlorosis and withering 7 days following the initiation of both salt and drought treatments. Conversely, in transgenic lines subjected to the same treatment, these morphological changes were greatly reduced compared to Col-0 plants (**Figures 7A–D**). ROS accumulation was also altered in transgenic leaves following osmotic stress treatment compared to untransformed controls, whereby transgenic lines were found to accumulate significantly lower levels of $O_{2}^{–}$ than Col-0 (**Figure 7E**). Furthermore, the water loss rate of detached rosette leaves from 4-week old transgenic plants were significantly reduced compared to Col-0 controls (**Figure 7F**), which indicates that transgenic lines were less affected than Col-0 by osmotic stress.

**FIGURE 8:**
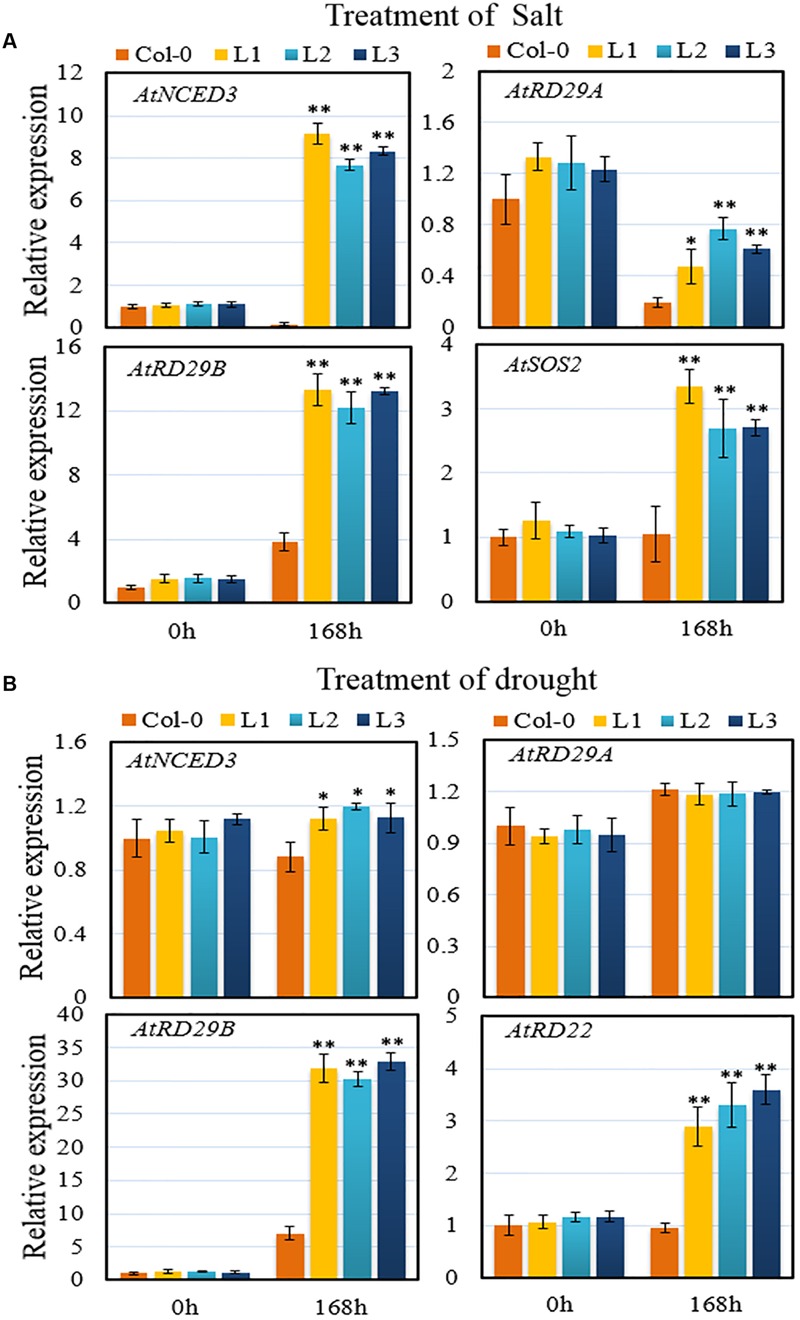
**Assessment of the expression of stress-related genes. (A,B)** The relative expression levels of osmotic stress-responsive genes were assayed in leaf tissue via qPCR 7 days following initiation of salt **(A)** and drought **(B)**. Data represent mean values ± SD from three independent experiments. Asterisks indicate significant differences between Col-0 and transgenic lines as determined by Student’s *t*-test (^∗^0.01 < *P* < 0.05; ^∗∗^*P* < 0.01).

To investigate the defense response pathways behind *VqSTS21*-mediated osmotic stress tolerance in *Arabidopsis*, we analyzed the expression of various genes in 5-week old transgenic and Col-0 plants 7 days after the initiation of salt and drought treatments (**Figures 8A,B**). Since ABA-mediated signaling is known to be one of the most important aspects of abiotic stress tolerance in plants (Zhang et al., 2012), we focused on the expression of genes known to be involved in this pathway. Genes included *AtRD29A* and *AtRD29B*, which are key downstream genes in the ABA-mediated response pathway, *AtRD22*, which is responsive to dehydration and is also involved in the ABA signaling pathway in response to abiotic stress (Huang et al., 2012), and 9-*cis*-epoxycarotenoid dioxygenase-3 (*AtNCED3*), which is an indicator of ABA biosynthesis (Iuchi et al., 2001). Following salt treatment, expression levels of *AtNCED3, AtRD29A* and *AtRD29B* were significantly higher in *VqSTS21* transgenic plants than in Col-0 (**Figure 8A**). Furthermore, the transcript level of salt overly sensitive-2 (*AtSOS2*), which is a component of the salt overly sensitive (SOS) pathway (Zhu, 2002), was also significantly enhanced in transgenic lines compared to Col-0 plants (**Figure 8A**). This suggests that along with ABA-mediated signaling, the SOS pathway may also play a role in the enhanced salt stress tolerance seen in our *VqSTS21* transgenic lines. In the case of drought stress, while the expression of *AtNCED3* and *AtRD29A* remained unaltered, *AtRD29B* and *AtRD22* expression was significantly increased by 4.5-fold and 3.4-fold in transgenic plants compared to control plants, respectively (**Figure 8B**).

## Discussion

There has been much interest in recent years concerning the biosynthesis of resveratrol, due to both its important medicinal properties (Aggarwal et al., 2004; Shankar et al., 2007) and its function in plant disease resistance (Hain et al., 1993; Jeandet et al., 2002). As such, studies concerning the generation of transgenic plants that produce this valuable stilbenoid via the heterologous expression of *STS* are accumulating. To date, the heterologous expression of *STS* has been successfully achieved in numerous plant species, including tobacco (Hain et al., 1993), tomato (Thomzik et al., 1997), rice (Stark-Lorenzen et al., 1997), lettuce (Liu et al., 2006), papaya (Zhu et al., 2004), alfalfa (Hipskind and Paiva, 2000), *Arabidopsis* (Yu et al., 2006; Liu et al., 2011), hop (Schwekendiek et al., 2007), wheat and barley (Leckband and Lorz, 1998), with increased disease resistance evident in each case. For example, transgenic rice bearing a grape *STS* gene displayed improved resistance to *Pyricularia oryzae* (Stark-Lorenzen et al., 1997), alfalfa expressing a peanut *STS* gene exhibited enhanced resistance to *Phoma medicaginis* (Hipskind and Paiva, 2000), and a resveratrol synthase gene (*PcRS*) from *Polygonum cuspidatum* restricted *Colletotrichum* spore production in transgenic *Arabidopsis* (Liu et al., 2011). In this study, we generated transgenic *Arabidopsis* lines that heterologously expressed an *STS* gene (*VqSTS21*) from Chinese wild *V. quinquangularis* cv. “Shang-24”, which is known to contain high levels of resveratrol and is naturally highly resistant to PM (Schnee et al., 2008), and assessed their tolerance to various types of biotic and abiotic stress.

It has been previously reported that *Arabidopsis* transformed with either a Sorghum *STS* gene or the *Polygonum cuspidatum PcRS* (resveratrol synthase) gene produced *cis*- and *trans*-piceid, respectively, at high concentrations (Yu et al., 2006; Liu et al., 2011). Similarly, hop and kiwi transformed with grape *STS* genes were also found to generate relatively high levels of piceid (Kobayashi et al., 2000), which implies that this may be a common phenomenon in *STS* transgenic plants and correlates well with the fact that *trans*-piceid was the major stilbene produced in the *VqSTS21* transgenic lines produced in this study (**Table 1**). Since piceid is derived from resveratrol via glycosylation, it has been suggested that resveratrol produced by heterologous *STS* genes is largely metabolized into piceid by endogenous glycosyltransferases (Kobayashi et al., 2000; Yu et al., 2006; Liu et al., 2011). Indeed, we failed to detect any resveratrol in our transgenic lines, which may be attributable to very efficient conversion into piceid within the plants and/or the method used for stilbenoid detection.

Plants defend themselves against different pathogens via the networking of several phytohormone-mediated signaling responses (Verhage et al., 2010; Derksen et al., 2013). Biotrophic pathogens generally induce the SA-mediated defense response, which activates various downstream physiological immune responses such as programmed cell death and ROS accumulation (Glazebrook, 2005; Guo et al., 2016). Semi-biotrophic pathogens, which depend on a living host for their nutrition early during infection, but rely upon dead host tissue later in the infection cycle, appear to induce both SA- and JA-mediated signaling responses (Song et al., 2015). In this study, the heterologous expression of *VqSTS21* in *Arabidopsis* led to enhanced resistance to the biotrophic pathogen, PM (**Figure 2**), which correlates well with previous research in which the *VqSTS5* gene from *V. quinquangularis* has been found to improve resistance to PM in transgenic *V. vinifera* plants (Cheng et al., 2016). Furthermore, our transgenic lines also exhibited reduced severity and spread of the semi-biotrophic *Pst* DC3000 compared to untransformed controls (**Figure 4A**). In both cases, this lessening of disease symptoms seen in transgenic lines also manifested as increases in programmed cell death and ROS accumulation (**Figures 2B,C** and **4B,C**), which may play a role in restricting disease spread in these lines.

Interestingly, *VqSTS21* transgenic *Arabidopsis* lines were also found to express several genes required for SA-mediated signaling at significantly higher levels than untransformed controls in response to inoculation with the biotrophic PM (**Figure 2D**), and both SA- and JA-mediated signaling in response to *Pst* DC3000 (**Figure 4D**). Taken together, these results suggest that heterologous expression of the grape *STS* gene improves resistance to biotrophic and semi-biotrophic pathogens at least in part through an enhancement of SA and/or JA-mediated pathways.

While stilbenoids have been shown previously to provide a positive effect on resistance to *B. cinerea* (Sbaghi et al., 1995; Adrian and Jeandet, 2012; Hatmi et al., 2014), reports exist in which resistance to this pathogen has not been achieved through the heterologous expression of *STS* genes (Thomzik et al., 1997). These latter findings correspond with our results, whereby *VqSTS21* transgenic *Arabidopsis* did not provide enhanced resistance to *B. cinerea* compared to untransformed controls, and instead increased their susceptibility (**Figure 3A**). Since *B. cinerea* is a necrotrophic fungus, and plants defend against such pathogens by inducing the JA signaling pathway (Grant and Jones, 2009; Verhage et al., 2010; Derksen et al., 2013), one would expect an augmentation of this response to be involved in improved resistance. We noted up-regulation of genes involved in SA-mediated signaling in *VqSTS21* transgenic lines compared to untransformed controls following inoculation with *B. cinerea*, and an inhibition of genes involved in the JA-signaling pathway (**Figure 3D**), which could very well be responsible for the increased susceptibility of transgenic lines to this pathogen. Interestingly, it has been suggested previously that SA- and JA-mediated signaling pathways are antagonistic (Derksen et al., 2013; Shim et al., 2013), and if plants defend against a particular pathogen via the SA-dependent pathway first, then JA-signaling is inhibited. These results suggest that heterologous expression of the grape *STS* gene improves resistance to biotrophic and semi-biotrophic pathogens, but not necrotrophic organisms.

In addition to its role in defense against particular plant pathogens, there is some evidence that stilbenoids could also contribute to improved tolerance to abiotic stress. For example, osmotic stress (Deis et al., 2011; Hatmi et al., 2014) and exogenous application of ABA (Luan et al., 2014), which is known to contribute to osmotic stress tolerance, increased the accumulation of stilbenes in wine grape. Furthermore, citrus seedlings treated with exogenous resveratrol exhibited reduced NaCl-derived membrane permeability and MDA accumulation (Kostopoulou et al., 2014), which correlates well with the fact that *VqSTS21* transgenic *Arabidopsis* lines displayed decreased MDA content in response to osmotic stress (**Figure 6F**). We also examined other physiological parameters known to be indicators of stress responsiveness, including chlorophyll content (Tu et al., 2016) and cell membrane integrity (Bajji et al., 2002). While chlorophyll content was determined to be significantly higher in *VqSTS21* transgenic lines than untransformed controls following osmotic stress, electrolyte leakage was significantly reduced in transgenic lines (**Figure 6F**). These results suggest that heterologous expression of *VqSTS21* yields plants that are better able to withstand drought and salinity as evidenced by less chlorosis and cell membrane damage, which means cellular ion concentrations, would be maintained to a greater extent under stress conditions. Similarly, the application of resveratrol has been found previously to reduce the accumulation of H_2_O_2_ in leaves and restore loss of photosynthesis induced by NaCl treatment (Kostopoulou et al., 2014), which is reminiscent of the diminished $O_{2}^{–}$ accumulation noted in *VqSTS21* transgenic lines compared to untransformed controls in response to osmotic stress in this study (**Figure 7E**). These findings provide further support for a role of plant stilbenoids in the defense against osmotic stress.

Although the ABA-dependent signaling pathway is known to play an indispensable role in plant resistance to abiotic stress (Sreenivasulu et al., 2012), and a recent study has demonstrated that ABA affects phenolic compounds in grape (Yamamoto et al., 2015), the precise relationship between *STS* and ABA-mediated signaling is unclear. Intriguingly, we found the expression of various ABA-responsive genes to be significantly up-regulated in *VqSTS21* transgenic *Arabidopsis* lines compared to untransformed plants under osmotic stress (**Figure 8**). These results suggest that the regulation of these ABA-responsive genes by *VqSTS21* or its products may contribute to the enhancement of osmotic stress resistance seen in these lines. The fact that we also noted significantly longer roots in *VqSTS21* transgenic seedlings in response to ABA treatment than untransformed controls (**Figure 6D**) further corroborates a role for the ABA-mediated defense signaling pathway in these lines. In addition, the expression of the SOS pathway gene, *AtSOS2*, which is essential for the reestablishment of cellular homeostasis under salt stress (Zhu, 2002), was up-regulated in our transgenic *Arabidopsis* lines compared to untransformed controls in response to salt stress (**Figure 8A**). This suggests that along with ABA-mediated signaling, the SOS pathway may also be playing a role in the enhanced salt stress tolerance seen in our *VqSTS21* transgenic lines.

In summary, plants are complex organisms that protect themselves from biotic and abiotic stress through networks of defense-related genes (Reuber et al., 1998; Verhage et al., 2010; Guo et al., 2016). In this study, we demonstrate that the introduction of the *VqSTS21* gene from *V. quinquangularis* into *Arabidopsis* promotes the SA-mediated signaling pathway to improve disease resistance to the biotrophic PM, and both SA- and JA-mediated signaling pathways to enhance resistance to the semi-biotrophic pathogen *Pst* DC3000. Conversely, the JA-mediated signaling pathway is suppressed in these lines in response to *B. cinerea* infection, resulting in increased susceptibility to this necrotrophic pathogen. We also found that tolerance to osmotic stress was enhanced in *VqSTS21* transgenic lines; a finding that appears to result, at least in part, from up-regulation of genes involved in ABA signaling. The nutrient ratio, in terms of N, P and K, in matrix soil is about 45: 9: 27, which may be the optimal nutrients ratio for plants growth; however, the ratio in MS medium was very different and reached about 60: 1: 20, this difference may cause the imbalance of nutrient on plant growth. MS medium is the most widely applied and recognized media to cultivate plants *in vitro*, but the better way to cultivate plants *in vitro* may need to be explored and developed. Our analysis of transgenic *Arabidopsis* lines that heterologously express *VqSTS21* provides new insight into the role of *STS* in stress response and a framework for future research in this field, which will almost certainly involve overexpression of this gene in grape.

## Author Contributions

XW and LH designed the experiments. LH, and SZ conducted the experiments. XW, XY, and JY supplied reagents/materials/analysis. XW and YW provided guidance throughout the entire study. LH, SS, and XW wrote the manuscript. All authors approved the final manuscript.

## Conflict of Interest Statement

The authors declare that the research was conducted in the absence of any commercial or financial relationships that could be construed as a potential conflict of interest.
