# Supplementary material for: Expression of the Grape VqSTS21 Gene in Arabidopsis Confers Resistance to Osmotic Stress and Biotrophic Pathogens but Not Botrytis cinerea
Source: Front Plant Sci. 2016 Sep 15;7:1379. doi: 10.3389/fpls.2016.01379 (PMC5024652; doi:10.3389/fpls.2016.01379)
Supplement: Supplementary file 1 [file Data_Sheet_1.DOC]

**Expression of the grape *VqSTS21* gene in Arabidopsis confers resistance to osmotic stress and powdery mildew but not *Botrytis cinerea***

**Li Huang^1, 2^, Songlin Zhang^1, 2^, Stacy D. Singer^3^, Xiangjing Yin^1, 2^, Jinhua Yang^1, 2^, Yuejin Wang^1, 2^, and Xiping Wang^1, 2^***

Supplementary Data

Supplementary Tables

**Table S1. Primers used for semi-quantitative RT-PCR**

| Genes | Forwards primers | Reverse primers | Product Length  (bp） |
| --- | --- | --- | --- |
| *VqSTS5,6* | GTGGGGCTACAAATTGAGTGAAAG | GGCGAAACTGTAAGAACTTGATGTC | 215 |
| *VqSTS7* | GGAGGGATTGTAATTTAGTGATCGT | CGCCAAGAACTTGAAGTCTCACT | 77 |
| *VqSTS9* | CCCTATCATAAAATTGAGGGATTG | GGGCATTTCAAGAATATAAAACAA | 112 |
| *VqSTS10* | CGATCATAACATTGAGGGATTGTAG | GGGGATACAACTTTTCAGGAAAC | 126 |
| *VqSTS15,21* | CGCCCTAAATCTTTTATTCCTATCC | CCCACAGAATGACAAGTACTTGCA | 116 |
| *VqSTS16,22* | CCTTCTGAAACTGCTTTGGACTCT | GGGTTGCGATAACTTCGTTATTGT | 670 |
| *VqSTS17,23,24* | CGGGTTTGATATCTGAAAACATAGAG | GGGAAGATGATTTCATGGTGATATATC | 453 |
| *VqSTS19,20,28,30* | CCCGTGAAATGATGGGAAAAGT | CGAACTGTAGAGTACCCCACCTGAA | 271 |
| *VqSTS27,29* | CCGCCCTATTATGTTGAATAGGAGT' | GGGTTTAGGGAATCTTCAAGATGAT | 190 |
| *VqSTS31* | CGGGGTTATTTATCTCCTAAACTAAT | CCGTTTAATTTGAGCTCACCAAG | 115 |
| *VqSTS32* | CAAACCATGAGAGTCCAAGTTCCC | GCGACACGTTGGTGTTCAAGTAGA | 216 |
| *VqSTS35* | TGCTGCATAGCGTTCCTCCA | CCCCACATGAACACAACATCAATA | 284 |
| *VqSTS36* | CGGGTATAAATTAAGTGAAGGGGAA | GGGGGGATAATGAAACAGTGAGATA | 214 |
| *VqSTS37* | CCCATAGAGAAATGCTTGACCCA | CCGACTTGATTACAAGCCAAATTTA | 483 |
| *VqSTS38* | TGCCACGGGTACAAATTGAGTTA | GGAAGCCCTCCAGCAATCAGT | 180 |
| *VqSTS39,43* | CGCCTATCGAAACTGTTGTGCTAC | CCCCCTTGAAACTCATCTTTTTAAT | 235 |
| *VqSTS41,45* | CCCGTATTTCATCACATTGGTAA | GGCAACTTGAAACTCATCTTCTTA | 96 |
| *VqSTS42* | CCCCAATTGATAAAACTCTTGTAGTA | CCCCTTTTAGTTTGAGCTAATCAC | 105 |
| *VqSTS46* | GGGGGGTTACAAATTAAGAGCAATA | GGGTGACTCAGGTACAAATCCAAAT | 159 |
| *VqSTS47* | CCCGTGAGGTAAAGAAGAATGGTC | GCCGCAGTCTAACAATGACTTGAA | 138 |
| *VqActin1* | GATTCTGGTGATGGTGTGAGT | GACAATTTCCCGTTCAGCAGT |  |

**Table S**2. Primers used for qRT-PCR

| Genes | Forwards primers | Reverse primers |
| --- | --- | --- |
| *AtPR1* | AACTACGCTGCGAACACGTG | TCACTTTGGCACATCCGAGTC |
| *AtICS1* | CTTCCGTGACCTTGATCCTTTCT | CAGCGATCTTGCCATTAGGATC |
| *AtPDF1.2* | GAAGCACAGAAGTTGTGCGA | TGTAACAACAACGGGAAAATAAACA |
| *AtLOX3* | TCTCCGTACAACAAGCGTTGG | GCGTCCGTCTAGCGCATTAAT |
| *AtSOS2* | ATTGAGGCTGTAGCGAAC | GGTATTCCTTCTGTTGCC |
| *AtRD22* | GGTTCGGAAGAAGCGGAGAT | AGTGGAAACAGCCCTGACGT |
| *AtRD29A* | AAGCAATGAGCATGAGCAAG | GGAAGACACGACAGGAAACAC |
| *AtRD29B* | ACGACGGAAACATCGGACT | CTTCACCACCAGGAGCAAA |
| *AtNCED3* | TTGATGCTCCAGATTGCTTC | GTCCACAGAAAAGCATAGCAG |
| *AtActin* | AGTGTCTGGATCGGTGGTTC | CCCCAGCTTTTTAAGCCTTT |
