# Supplementary figures and images for: Expression of the Grape VqSTS21 Gene in Arabidopsis Confers Resistance to Osmotic Stress and Biotrophic Pathogens but Not Botrytis cinerea
Source: Front Plant Sci. 2016 Sep 15;7:1379. doi: 10.3389/fpls.2016.01379 (PMC5024652; doi:10.3389/fpls.2016.01379)

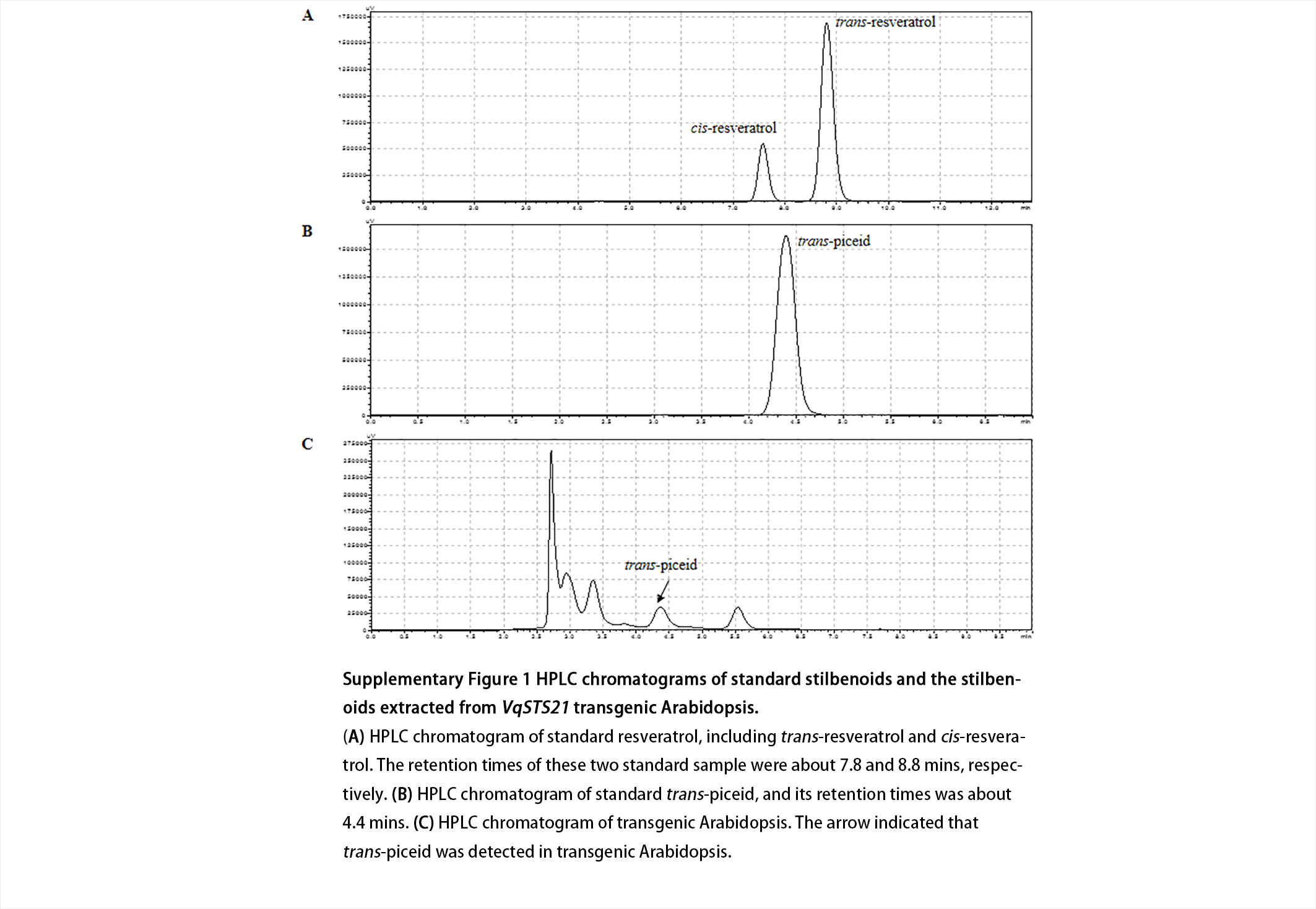

Supplement: Supplementary file 2 [file Image_1.TIF]
